# Nanomedicine And Nanotheranostics: Special Focus on Imaging of Anticancer Drugs Induced Cardiac Toxicity

**DOI:** 10.7150/ntno.96846

**Published:** 2024-06-03

**Authors:** Aseem Setia, Randheer Reddy Challa, Bhaskar Vallamkonda, Phanikumarreddy Satti, Abhishesh Kumar Mehata, Vishnu Priya, Senthil Kumar, Madaswamy S. Muthu

**Affiliations:** 1 Department of Pharmaceutical Engineering and Technology, Indian Institute of Technology (BHU), Varanasi-221005, India.; 2 Department of Pharmaceutical Science, School of Applied Sciences and Humanities, VIGNAN's Foundation for Science, Technology & Research, Vadlamudi-522213, Andhra Pradesh, India.; 3 Department of Chemistry, Acharya Nagarjuna University, Guntur- 522510, Andhra Pradesh, India.; 4 Pharmacy Services, Homi Bhabha Cancer Hospital & Mahamana Pandit Madan Mohan Malaviya Cancer Centre, Varanasi-221005, India.

**Keywords:** Cardiotoxicity, anticancer drugs, molecular mechanism, nanomedicine, nanotheranostics

## Abstract

Cardiotoxicity, the often-overlooked second leading cause of death in cancer patients, has been associated with certain anticancer drugs. These drugs can induce cardiac damage through various pathways, and their adverse effects on the heart are not fully understood. Cardiotoxicity is a major issue in cancer treatment, particularly with chemotherapeutics, because it can cause cardiac dysfunction such as hypotension, heart failure, and even death. Doxorubicin, 5-fluorouracil, and trastuzumab, all of which are very potent anticancer drugs, are known to cause cardiotoxicity. When it comes to lowering cardiotoxicity and alleviating the harmful effects of chemotherapy medications, nanomedicine has the potential to transport therapeutic molecules. Nanotheranostics offers novel options for identifying and treating cardiotoxicity resulting from a wide range of substances, including anticancer medications. Additionally, theranostics platforms such as micellar systems, carbon-based nanomedicine, solid lipid nanoparticles, polymeric nanoparticles, and liposomes can transport chemotherapeutic medications while minimising their cardiotoxicity. The present level of understanding of the molecular and cellular processes that lead to cardiotoxicity in reaction to both traditional chemotherapy and targeted drug delivery systems is summarised in this article. This review delves into nanomedicine and nanotheranostics, with an emphasis on reducing anticancer medication-induced cardiac toxicity. Nanotheranostics provide potential solutions for early diagnosis and tailored therapy of heart injury by combining diagnostic and therapeutic capabilities into nanomedicine.

## Introduction

The heart is a very important part of the human body, responsible for circulating blood throughout the body, transporting oxygen and nutrients to the cells, and eliminating waste products such as carbon dioxide. There is no cell division or regeneration in cardiomyocytes. Thus, injury to the cardiomyocytes has a detrimental effect on heart function. Heart problems caused by anticancer medicines are a growing worry for oncologists 1. The prevalence of cancer increases with age, peaking between 40 and 80 years old 2. Oncologists face a significant challenge in reducing the risk of deadly cardiac dysfunction caused by antineoplastic therapy while maintaining it in the face of minimal or no cardiovascular adverse effects 3. Since the development of antineoplastic drugs, the outlook for cancer patients has drastically improved. However, many chemotherapy drugs cause cardiotoxicity, which manifests itself as either immediate or delayed cardiac dysfunction 4. Despite the lack of consensus surrounding its definition, cardiotoxicity is typically understood in clinical practice to mean a decrease in left ventricular ejection fraction (LVEF). Differences in LVEF threshold alterations were used to determine cardiotoxicity by different groups and clinical committees 5. The incidence of cardiac dysfunction following treatment with anthracyclines ranges between 2% and 48% 6. Among the several types of chemotherapy, this one is most often associated with concerns about cardiotoxicity. A reduction in LVEF may not be a significant element to detect a subclinical myocardial dysfunction that consequently emerges into symptomatic congestive heart failure (CHF), despite the fact that using this method of assessment is common clinical practice since it helps to detect cardiotoxic side effects before they cause permanent heart damage or heart failure (HF) 7.

Despite decades of research and attempts to diminish the cardiac adverse effects of standard chemotherapeutic moieties, there are still many unknowns about the molecular causes related to cardiotoxicity. This complexity has only increased with the arrival of new cardiotoxic medicines on the market 8. Doxorubicin (DOX)-induced cardiotoxicity is typically characterized by classical mechanisms, including DNA damage, lipid peroxidation, cardiomyocyte apoptosis, protein modification, oxidative stress, etc. It has been postulated that similar routes underlie the cardiotoxicity of daunorubicin, epirubicin, and idarubicin, even if mitoxantrone has certain unique actions with relevant clinical repercussions 9. Even though docetaxel's cardiotoxicity is much less common than anthracyclines, it is nevertheless a worry, and the use of anthracyclines in combination has been upraised. Direct cytotoxic damage and lipid peroxidation have both been proposed as possible contributors. Disrupted microtubule activity in cardiomyocytes can lead to mitochondrial injury and altered metabolism by preventing the mobilization and storage of fatty acids (FA) obtained from cytosolic pools (such as lipid droplets). Though melphalan's cardiotoxicity has been established, no effective mitigation strategies have been identified 10.

A compelling framework for tackling cardiotoxicity, a major issue in drug development and clinical practice, is offered by nanotheranostics, a combination of nanotechnology and theranostics 11. By combining diagnostic and therapeutic capabilities, nanotheranostic systems make it possible to detect and treat cardiac toxicity all at once. Common components of such platforms include nanoparticles designed to transport diagnostic imaging agents and therapeutic intervention agents 12. When it comes to cardiotoxicity, there are nanotheranostic platforms that can be developed to identify changes in the cardiac system. One example is the use of heart-specific targeting ligands on nanoparticles, which allow them to aggregate exclusively in cardiac tissues and improve diagnostic specificity and sensitivity 13. To enable non-invasive, high-resolution monitoring of heart function, these nanoparticles can carry imaging agents like contrast agents for MRI, positron emission tomography (PET), or optical imaging using fluorescent dyes. Nanotheranostic platforms also allow for the direct delivery of therapeutic drugs to the injured cardiac tissues, allowing for targeted, localised treatment with minimal systemic adverse effects 14, 15. Potential therapeutic payloads encompass antioxidants, cardioprotective drugs, or compounds that target certain pathways associated with cardiotoxicity, including oxidative stress or apoptosis. The ability to monitor treatment responses in real-time and change therapeutic methods as needed is made possible by nanotheranostic systems, which combine diagnostic and therapeutic capabilities. This allows for personalised and effective control of cardiotoxicity 16. This review provides the pathophysiology of anticancer drug-induced cardiac dysfunction. Additionally, this review focuses on the promising field of nanomedicine and nanotheranostics for improving diagnostics, targeting therapy, and real-time imaging which shows the cardiac toxicity caused by anticancer drugs.

## Anticancer drug-induced cardiac dysfunction

### Pathophysiology of cardiac dysfunction

**Figure 1:**
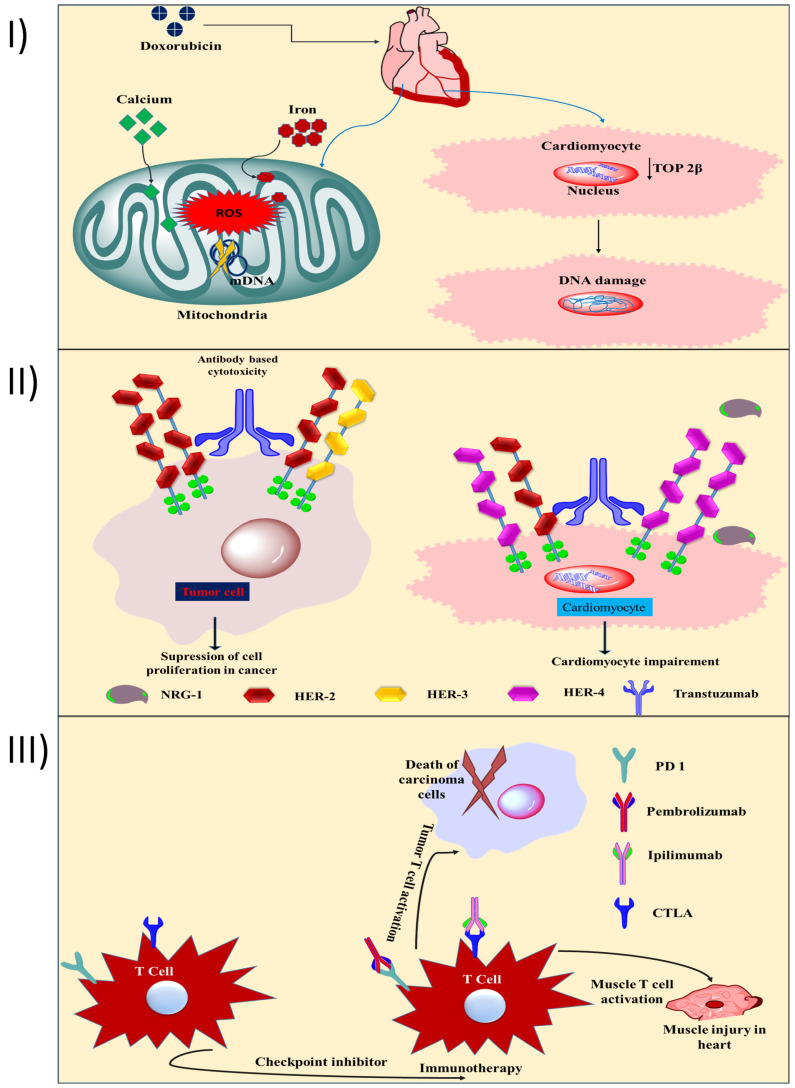
** I)** Toxic effects on the heart are a result of anthracycline's ability to inhibit TOP 2β and generate ROS. Reducing TOP 2β activity in cardiomyocytes leads to DNA intercalation and cardiac DNA damage. The mitochondrial DNA is damaged when ROS are produced in excess (mitochondrial DNA). DOX therapy causes cardiotoxicity, in part due to intracellular Ca and Fe accumulation; **II)** As HER-2 is overexpressed in breast cancer and is directly responsible for the tumor's growth, trastuzumab encourages its dimerization. Trastuzumab inhibits HER-2 homodimerization and heterodimerization with HER-3, hence preventing cancer cell proliferation (left side). Trastuzumab blocks the action of neuregulin, preventing the protective dimerization of HER-2 and cardiomyocytes, leading to mitochondrial malfunction and promotes cellular damage; **III)** Ipilimumab, blocks antitumor T cell responses by turning off a negative regulator of T cell activation, and pembrolizumab, an inhibitor of PDL-1, allows infiltrating T cells to do their function once they've cleared the tumor's microenvironment. T-cell responses in the heart and muscle are boosted by ipilimumab and nivolumab also contribute to autoimmune cardiotoxicity.

Two distinct forms of cardiac myopathy caused by cancer drugs have been identified. Anthracyclines, mitoxantrone, and cyclophosphamide produce type 1 cardiotoxicity, which is irreversible and results from oxidative stress on cardiac myocytes and mitochondrial malfunction, leading to morphological abnormalities. Trastuzumab and tyrosine kinase inhibitors (TKIs) are responsible for type 2 cardiotoxicity, which is transient heart damage that prevents cardiomyocytes from thriving and repairing themselves but does not alter their structure 17, 18. The cardiotoxicity of anthracyclines is highly dose-dependent; for example 2.2% of DOX recipients develop HF with a continuous dosage of 300 ~ <550 mg/m^2^, and this incidence rises to 26-30% at total doses of 500-800 mg/m^2^ 19. Patients who are dosed with DOX have a mortality rate from heart failure of between 27 and 60% 20. However, another study found that the risk of HF was much lower for patients treated with epirubicin or idarubicin compared to patients treated with DOX (4% vs. 36%, respectively) at a dose of 500-550 mg/m^2^. Inhibition of topoisomerase 2β (TOP 2β) and reactive oxygen species (ROS) are the primary causes of doxorubicin-induced heart injury 21. In cardiomyocytes, TOP 2β is overexpressed. Doxorubicin binds to TOP 2β and DNA, forming a stable compound. DNA damage and subsequent cell death are the results of this complex. Free radical injury (Fig. 1I) and DNA damage are both triggered by DOX, and both have been linked to the increased formation of reactive oxygen and nitrogen species (RNS). In addition to iron build up in the mitochondria and calcium overload in the sarcoplasmic reticulum, two other mechanisms for DOX-induced heart injury contribute to a lack of protein synthesis 22. According to another study, the carcinogenic effects of DOX come from its metabolite, doxorubicinol. When compared to DOX, doxorubicinol is more effective at blocking calcium channels 23. Human epidermal growth factor receptor 2 (HER-2) is a protooncogene that is strongly expressed in 20-30% of breast tumors and is linked to a poor prognosis, an increased risk of metastasis, and resistance to treatment for breast cancer 24. Cell survival and the activation of protective pathways are induced by HER-2, HER-4/HER-4 homodimerization, and HER-2/HER-2 heterodimerization in the heart, especially during cardiac stress (Fig. 1II) 25. Neuregulin (a dimerization trigger) inhibition in the heart disrupts HER-4/HER-4 homodimerization and HER-4/HER-2 heterodimerization in cardiomyocytes, resulting in mitochondrial dysfunction-mediated cell death. The mechanism of cardiotoxicity caused by tyrosine kinase inhibitors (TKIs) has been documented previously 26. HF and left ventricular dysfunction (LVD) are examples of the cardiotoxicity associated with small molecule-based TKI. Cell death and mitochondrial dysfunction are the results of imatinib's cytotoxicity. The drug also triggers the endoplasmic reticulum (ER) stress response 27. The mechanism of action of 5-fluorouracil (5-FU) that causes cardiotoxicity has been the subject of recent in-depth reviews 28. Thrombosis caused by endothelial injury and higher metabolism both contribute to 5-FU's cardiotoxicity induction mechanism, which ultimately results in energy depletion, ischemia, and cellular destruction. Additionally, 5-FU reduces red blood cells' oxygen-carrying capacity, triggers oxidative stress, and promotes coronary spasms, all of which result in myocardial infarction 28. When applied to cancers with a dismal prognosis, immunotherapy, one of the newest forms of cancer treatment, can be a game-changer. Immunotherapy is preferred over small-molecule chemotherapies because of its lower risk for patients. Avelumab, pembrolizumab, ipilimumab, and nivolumab are ICI that cause autoimmune cardiotoxicity 29. Preclinical data shows that animals lacking the genes for programmed cell death 1 (PD 1) and cytotoxic T lymphocyte antigen (CTLA) began to die between the third and fifth week due to cardiac problems 30. Cardiotoxicity is induced by pembrolizumab (a PD-1 inhibitor) due to an increase in intracellular calcium and a subsequent decrease in cardiomyocyte viability. When T cells target an antigen represented by the neoplasm and muscle fibres, myocarditis might develop due to the lack of specificity of the heightened T cell activation in cardiac and skeletal muscle (Fig. 1III). House *et al.,* reported a case study of a patient who had got acute MI shortly after taking vinca alkaloids. After undergoing chemotherapy that included vinca alkaloids, a 32-year-old man with stage IIIA nodular sclerosing Hodgkin's disease and no cardiac risk factors came out with chest pain. He had a myocardial infarction of the anterior wall. Angiography of the coronaries showed no evidence of severe blockage. Reduction in left ventricular ejection fraction and loss of anterior wall motion were confirmed by exercise stress testing using a gated scan. In rare cases, myocardial infarction has been documented as a side effect of vinca alkaloid-based anticancer treatments. Possible causes include hypercoagulable conditions, tumour invasion of the heart, and coronary artery spasm. The most likely cause is a spasm in the coronary arteries. The treatment entails removing the offending substance from the patient's system and providing supportive care 31.

### Chemotherapeutics with cardiac toxicity

Among the several classes of chemotherapeutic medicines used to treat cancer, anthracycline antibiotics are among the most potent. The various kind of cancers, such as small cell lung cancer, lymphoma, gastric cancer, sarcomas, and breast cancer are only a few of the malignancies that have been shown to be effective against anthracyclines. For instance, the incidence of heart arrest gradually increases from 2.2% to 7% at a dose above 550 mg/m^2^ while using an anthracycline 19, 32. This is known as dose-dependent heart failure and myocarditis. More than 40% of patients die within the first year of treatment due to cardiomyopathy. Daunorubicin causes cardiomyopathy in about 10% of individuals. One-sixth of patients treated with a combination of trastuzumab and anthracyclines developed severe HF. Compared to the occurrence rate linked with anthracycline therapy alone, this rate was significantly higher. Seventeen percent of 172 children receiving daunorubicin at a dose of 360-1260 mg/m^2^ developed cardiac problems. 5-FU is a highly effective anticancer medication frequently used to treat cancer of the stomach and intestines. These days, raltitrexed and other medications are being evaluated as alternatives because of the cardiotoxic effect they have on the body 33.

The cardiotoxicity profile of 5-FU has been extensively explored in a number of reviews. In 1997, researchers found that 1.9% of 5-FU individuals experienced symptomatic cardiac problems 34. A meta-analysis of data from various clinical trials revealed that 7.9 percent of individuals experienced cardiac problems. Approximately 44.9% of the patients also experienced arrhythmia, and 1.9% of those patients also experienced an ischemia event 35. Treatment with antibodies against CTLA-4 and PD-1 (immune-checkpoint inhibitor [ICI]) therapy has been linked to a wide range of cardiotoxic outcomes 36. Patients treated with pembrolizumab were reported in multiple studies to have developed autoimmune myocarditis and heart failure. Left ventricular dysfunction was seen in 79% (23 out of 30) of patients who received ICI, and death rates were 27% among this group. Patients with no other cardiac risk factors than hypertension were found to have developed myocarditis after receiving a combination of ipilimumab and nivolumab, according to a case report from 2016 37. Patients who received the combination of 3 mg/kg ipilimumab and 1 mg/kg nivolumab had myocarditis. The consequences of radiation therapy include pericarditis, constricted cardiomyopathy, hastened coronary heart disease, and valvular heart disease. The chance of having heart disease between 5-10 years following radiation therapy is 10-30% 38. Commonly used anticancer medications and their associated risks to cardiovascular health are summarized in Table 1.

#### Fluoropyrimidine-induced cardiotoxicity

The 2^nd^ most prevalent cause of chemotherapy-induced cardiotoxicity is fluoropyrimidines. The antimetabolite medication 5-FU and its precursor medicine capecitabine are highly effective in the treatment of certain malignancies, including those of the colon, breast, stomach, pancreas, prostate, and bladder 39. Although fluoropyrimidines have a high safety profile, 1-18% of people who take them will develop cardiovascular damage. Fluoropyrimidines can cause coronary artery spasm and myocardial ischemia, two cardiovascular adverse effects. Myocardial infarction, thrombosis of the coronary arteries, and processes occurring within cardiomyocytes are further potential reasons for cardiomyocyte loss and mortality 40. Cardiomyocytes, vascular smooth muscle cells, erythrocytes, and endothelial cells were all implicated as mediators of these deleterious effects. The chemical mechanism by which 5-FU causes vasoconstriction in vascular smooth muscle cells has been disclosed. Relative ischemia of the myocardium was induced by 5-FU, which was demonstrated to decrease the oxygen transport capacity of erythrocytes. It has been hypothesized that 5-FU injection causes an increase in ROS production in endothelial cells, which then causes cell senescence and death, which in turn causes a procoagulant state and acute thrombotic events. Finally, it has been proposed that fluoropyrimidines cause direct toxicity to cardiomyocytes. In fact, it has been shown that 5-FU promotes reactive oxygen species generation and induces apoptosis and autophagy in cardiomyocytes 41.

#### Doxorubicin-induced cardiotoxicity

Anthracycline antibiotic doxorubicin (DOX) was initially isolated from *Streptomyces peucetius* in the 1960s and utilised as a cytotoxic medication for the first time in 1969. Acute lymphoblastic leukaemia, paediatric leukaemia, lung cancer, lymphomas, and a wide variety of other metastatic cancers all respond very well to this extremely effective chemotherapeutic medicine. The drug's limited usefulness is due to the numerous adverse effects it causes, the most serious of which is cardiac damage. These adverse effects include suppression of the haematological system, gastrointestinal disorders, and baldness 42. Myocardial toxicity was shown to be present in over 30% of terminally ill cancer patients treated with high doses of doxorubicin over the course of more than a month. Various symptoms were present, including ventricular failure, a shortened QRS complex, cardiac dilatation, a rapid heart rate (150 beats per minute), and low blood pressure (70/50 mmHg) 43. Patients did not respond to inotropic medications or mechanical circulatory support. Histopathological findings included fewer myofibrils, a rearranged sarcoplasmic reticulum, vacuolized cytoplasm, inflamed mitochondria, and an increase in the frequency of lysosomes. Myocardial toxicities were observed in a variety of animal models after doxorubicin administration, including rats, mice, and rabbits. Doxorubicin administration reliably induced cardiomyopathy and cardiac failure in experimental animals across multiple studies 44.

#### Taxane-induced cardiotoxicity

Paclitaxel and other taxanes are antimitotic drugs that halt cell division by stabilising mitotic spindle microtubules. Cancers such as breast, lung, and ovarian are commonly treated with these chemotherapy medicines. However, taxane-based treatment regimens are not as effective as they could be, due to their severe toxicities. It has been observed that 2-4% of patients receiving a taxane experience cardiotoxic episodes. QT prolongation, bradycardia, and atrial fibrillation are all cardiotoxic consequences caused by exposure to taxanes 45. However, several suggestions about the cellular and molecular mechanisms behind taxane-induced cardiotoxicity have been postulated. Arrhythmia and other disturbances of the conduction system have been linked to hypersensitivity reactions with large histamine release. Consequently, anti-inflammatory (glucocorticoids) and anti-histamine (histamine receptor blockers) medications are recommended as preventative therapy for the treatment of taxane-induced cardiac anaphylaxis 46. The medication may be damaging cardiomyocytes by interfering with their subcellular organelles, according to a second theory. According to this theory, taxanes cause mitochondria in cardiomyocytes to produce more ROS, expand their permeability transition pore, and lose their membrane potential. Paclitaxel is a taxane that has been proven to increase the toxicity caused by anthracyclines. Indeed, greater HF episodes and increased histological changes of heart tissue, including severe necrosis, were seen in patients treated with paclitaxel and doxorubicin together. It was hypothesised that paclitaxel's involvement with the pharmacokinetics of doxorubicin's elimination was responsible for this impact. Neither docetaxel nor any other taxane has been shown to interact with doxorubicin, and neither drug increased the other's cardiac toxicity when used together 47.

#### Trastuzumab-induced cardiotoxicity

Patients with breast cancer with an elevated level of human epidermal growth factor receptor 2 (HER2, also known as ErbB-21) are treated with this monoclonal antibody. Although trastuzumab therapy has been shown to improve morbidity and mortality in breast cancer patients, it is important to monitor for major cardiac side effects. Trastuzumab is highly effective against HER2-positive breast cancers. However, studies using mutant mice have shown that ErbB-2 genes play a crucial role in postnatal cardiomyocyte function and development 48. Evidence suggests that ErbB-2 genes also play a role in cardiomyocyte maintenance and the inhibition of apoptosis, as well as in the normal functioning of the myocardium. These pathways include phosphoinositide 3-kinase, mitogen-activated protein kinase, and focal adhesion kinase. Therefore, trastuzumab's capacity to induce cardiotoxicity may have been related to its ability to disrupt ErbB signalling. However, individuals receiving concurrent anthracycline therapy had a higher chance of developing trastuzumab cardiotoxicity. The risk of cardiac toxicity and heart failure is raised when trastuzumab is used to inhibit ErbB2 signaling, as this increases the rate at which the anthracycline induces sarcomeric protein breakdown 49.

## Therapeutic potential of several nanomedicines in reducing cardiotoxicity

Numerous nanomedicines have shown encouraging results in lowering cardiotoxicity, opening up new possibilities for the treatment of cardiovascular disease. To minimize off-target effects and allow precision administration of cardioprotective medicines, tailored drug delivery methods can be employed using nanoparticles such as dendrimers, polymeric micelles, and liposomes 59. Carbon nanotubes, graphene oxide, and other carbon-based nanomedicine have special physicochemical features that make them good at reducing the damage to heart cells caused by oxidative stress. Nanoparticles made of metals, such as gold and silver, help to protect the heart since they are anti-inflammatory and antioxidative. In addition, the synergistic benefits of nanocomposites, which combine several nanomedicines, allow for greater therapeutic efficacy 60. Before clinical translation may occur, however, their biocompatibility, pharmacokinetics, and safety profiles over the long term must be thoroughly evaluated. Despite obstacles, there is significant hope for improving the treatment of cardiovascular illness and reducing cardiotoxicity through the therapeutic potential of nanomedicine 61. Th therapeutic potential of several nanomedicine in reducing cardiotoxicity shown in Table 2.

### Liposome-based nanomedicines in reducing cardiotoxicity

One of the most prevalent nanomedicines for chemotherapy drug delivery and cardiotoxicity reduction is lipid nanoparticles. Because of their simplicity of synthesis, long-term safety, and capacity to encapsulate hydrophobic and hydrophilic medications, lipid nanoparticles have attracted a lot of attention. Lipid nanoparticles have several uses, but the majority of the research on their usage has concentrated on delivering chemotherapeutic drugs and reducing cardiotoxicity 70. Liposomes are nanomedicine that has recently emerged as a promising tool in cancer treatment for the targeted delivery of chemotherapeutic medicines. There is hope that liposomes, which are nanoscale lipid vesicles, can reduce cardiotoxicity caused by several medicinal treatments. Their ability to encapsulate drugs makes it possible to distribute them specifically to heart tissues while reducing side effects. One way to lessen the likelihood of harmful cardiac consequences is to encapsulate cardiotoxic medications, like anthracyclines used in cancer treatment, within liposomes. The therapeutic potential of drugs can be further optimised by liposomal formulations, which increase their solubility, stability, and circulation time. Liposomes are a potential new direction in cardiovascular medicine because they allow for fine-grained regulation of drug release kinetics, providing a personalised strategy to reduce cardiotoxicity without sacrificing therapeutic efficacy 71.

**Figure 2:**
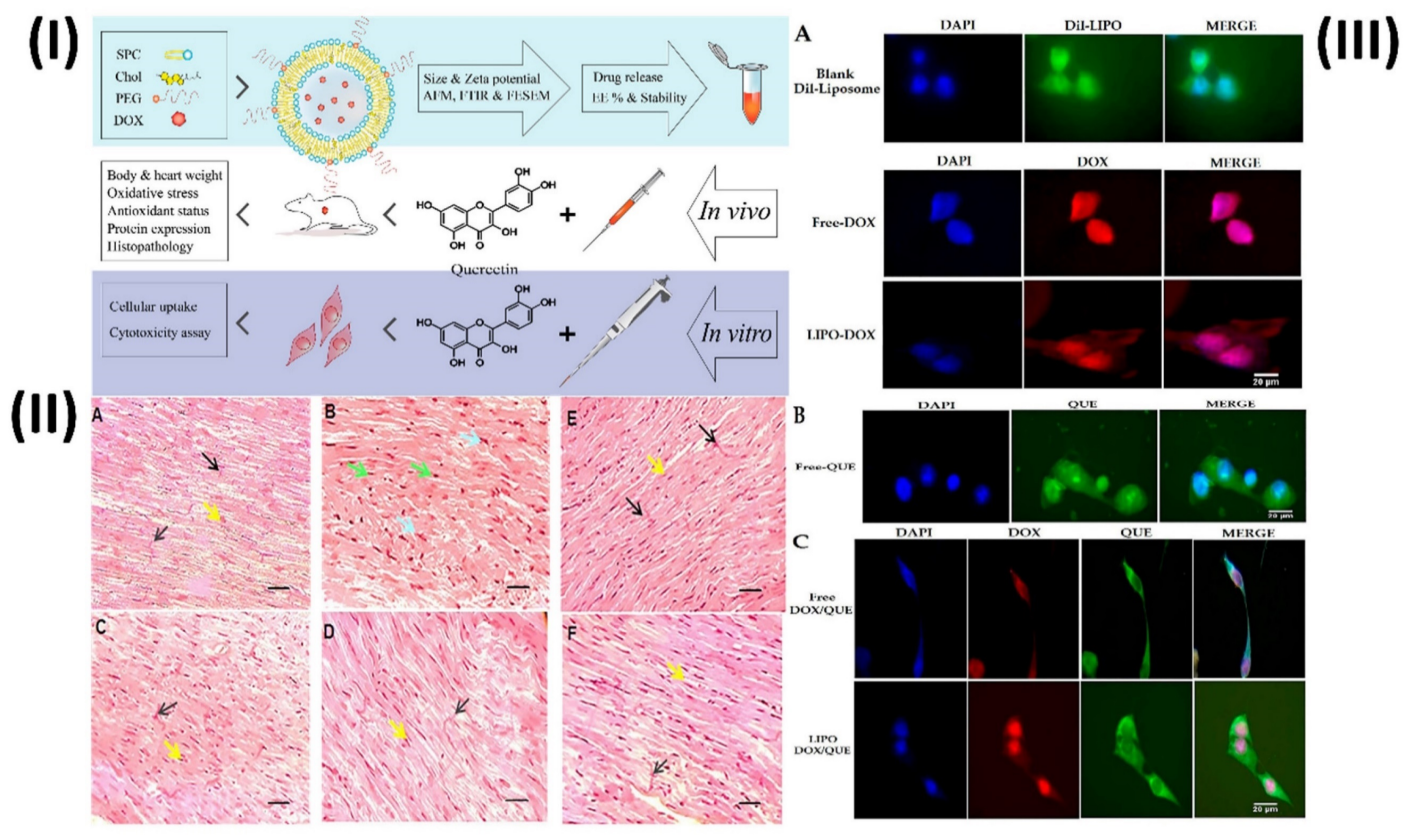
(**I**) A scheme of study steps; (**II**) Various groups of rats that had their left ventricles interfered on had their tissue stained with HE; (**III**) The cellular uptake of H9c2 cells, after being treated with (**A**) DIL-labeled liposome, free doxorubicin and liposomal doxorubicin, (**B**) free quercetin and (**C**) free doxorubicin and quercetin and liposomal doxorubicin and quercetin for 180 min. Reproduced with the permission from ref. 62. Fig. 1, Fig. 7 and Fig. 8 (MDPI©2023).

In a study, Dorostkar *et al.* formulated doxorubicin liposomes with desirable qualities using the thin-film method. Afterward, animal models were used to examine the effects of various substances. Measurements of cardiac enzymes, oxidative stress and antioxidant indicators, protein expression, and histological examinations were conducted to assess the therapies. In addition, H9c2 cells were used for cellular uptake and cytotoxicity assays. The developed liposomes had an encapsulation efficiency of approximately 85% and a mean size of 98.8 nm. Aside from being anionic and pH-sensitive, the developed liposomes exhibited a regulated release pattern and were very stable. By enhancing the expression of glutathione peroxidase, catalase, and superoxide dismutase enzymes in their left ventricles, rats were able to minimize weight loss, creatine kinase, lactate dehydrogenase, and malondialdehyde when liposomal doxorubicin and free quercetin were combined. Tissue damage and cell cytotoxicity were additional outcomes, as were changes in the expression of proteins such as NOX1, Rac1, Rac1-GTP, SIRT3, and Bcl-2. These findings demonstrate that therapies can improve antioxidant capacity, decrease cardiac tissue oxidative stress and apoptosis, and decrease the likelihood of problems (Fig. 2) 62.

Moreover, Batist *et al.* attempt to find out whether liposome-encapsulated doxorubicin and cyclophosphamide, when used as a first-line treatment for metastatic breast cancer (MBC), considerably decrease doxorubicin cardiotoxicity while maintaining comparable antitumor efficacy. The randomised controlled trial compared the efficacy of 60 mg/m2 of Myocet (M) with conventional doxorubicin (A) plus 600 mg/m2 of cyclophosphamide (C) every three weeks until disease progression or unacceptable toxicity was reached in 297 patients with metastatic breast cancer (MBC) who had not previously undergone chemotherapy for their disease. To determine cardiotoxicity, researchers used serial multi gated radionuclide angiography images to measure changes in left-ventricular ejection fraction, and CHF was also considered. Subjective tumour response rates (according to World Health Organisation standards), time to progression, and survival were used to evaluate the antitumor efficacy. Cardiotoxicity occurred in 6% of MC patients and 21% of AC patients (including 5 instances of CHF). With a hazard ratio of 5.04 the median cumulative doxorubicin dose at start was over 2,220 mg/m2 for MC compared to 480 mg/m2 for AC. Grade 4 neutropenia was also less common in MC patients. The effectiveness of MC in treating tumours was similar to that of AC: objective response rates were 43% and 43%, median time to progression was 5.1% and 5.5 months, median time to treatment failure was 4.6 and 4.4 months, and median survival was 19 and 16 months, respectively 63.

### Solid lipid nanomedicines in reducing cardiotoxicity

First generation lipid-based nanomedicine, solid lipid nanoparticles (SLNs) are stabilised emulsifier-formulated lipids, which are solid at body temperature. SLNs are smaller than a micron, or less than 1000 nm 72. Protecting drugs against environmental hazards is just one of many benefits; others include biocompatibility, biodegradability, the convenience of large-scale manufacture through the high-pressure homogenization technique, etc. The use of SLNs is gaining traction as a potential method to mitigate the cardiotoxicity of pharmacological formulations, especially those involving cancer treatments and medications for specific heart disorders 73. One of the many ways in which these nanomedicines can reduce cardiotoxic effects is by encasing the active medicinal components in a lipid matrix. As a first benefit, SLNs make it possible to regulate the release of medications, which means that they can be slowly and steadily delivered to specific tissues while reducing systemic exposure. The peak blood concentrations of medications, which are frequently linked to cardiotoxicity, can be mitigated with the aid of this controlled release profile 74. The second benefit of SLNs is that they allow for the delivery of medications to specific locations, such as cardiac tissues, within the body. The nanomedicine can minimise exposure to other organs and tissues by targeting ligands or peptides functionalized onto the surface of SLNs that identify receptors overexpressed in cardiac cells, allowing the nanoparticles to aggregate preferentially in the heart. In addition, the lipid matrix of SLNs provides a protective layer for the encapsulated medicines, preventing their metabolism and degradation, which could lead to cardiotoxicity 75. Zhang *et al.*, developed doxorubicin SLN for breast cancer. A pH-sensitive lipid conjugated with RGD and called RGD-HZ-GMS was produced in this study utilizing GMS as the lipid ingredient. A kind of SLNs called RGD-DOX-SLNs were created by encapsulating DOX with RGD-HZ-GMS. The anticancer impact of RGD-DOX-SLNs was tested using MCF-7 cells, which are breast cancer cells, and MCF-7/ADR cells, which are DOX resistant cells. Breast cancer model mice carrying MCF-7/ADR cells were used to assess *in vivo* tumor suspension and toxicity effects 64. This buffering effect aids in keeping the medication payload stable and effective throughout circulation and absorption by heart cells. Another benefit of SLNs is their ability to improve medication delivery to cardiac tissues due to their small size, which enhances cellular uptake. Because of this enhanced absorption, medication therapeutic efficacy can be enhanced while the total dosage can be reduced, which may reduce the risk of cardiotoxicity. Furthermore, SLNs can be modified to be more biodegradable and compatible with the body, reducing the risk of side effects and tissue harm when administered. It is possible to further enhance the safety profile of SLNs and make them appropriate for clinical applications by surface modifying them with biocompatible polymers or coatings 76.

To reduce DOX-induced cardiotoxicity, Zhang *et al.* construct an SLNs that is loaded with Resveratrol (Res). The Res-SLN was optimised using the central composite design/response surface approach after being created using the emulsification-diffusion method and sonication. High performance liquid chromatography was used to assess the drug loading and release profile, while dynamic light scattering and transmission electron microscopy were used for the morphological evaluation of the Res-SLN. In addition, they studied the impact of inhibiting DOX-induced cardiotoxicity in mice and found the Res distribution *in vivo* in rats. Res-SLN was effectively developed and fine-tuned to have a uniform particle size of 271.13 nm. Despite Res's poor solubility, the synthesised Res-SLN exhibited stability under storage and a sustained release profile. Compared to mice who experienced cardiac toxicity due to a single high-dose intraperitoneal injection of DOX, those who received Res-SLN treatment had significantly greater heart rates, ejection fractions, and fractional shortening. Furthermore, they measured the severity of cardiac ultrastructural abnormalities in mice. Protecting the myocardium and lowering DOX-induced cardiotoxicity are two of Res-SLN's therapeutic effects in mice 65.

In another study, Saad *et al.* employ supercritical antisolvent (SAS) technology to develop hesperidin (HES) loaded SLNs, which enhance the oral administration of HES. The encapsulation efficiency was measured at 87.6 ± 3.8% and the process parameters were fine-tuned to create HES-SLNs with a tiny size of 175.3 ± 3.6 nm. It was demonstrated by DSC and XRD that HES is distributed amorphously in SLNs. The aqueous solubility and apparent permeability of HES-SLNs were approximately 20 and 5 times higher, respectively, than those of HES. The bioavailability of HES from SLN formulation was roughly four times more than that of HES solution, according to rat pharmacokinetics. In comparison to the DOX group, HES-SLN considerably reduced DOX-induced cardiotoxicity by reducing cardiac troponin I and creatine kinase-muscle/brain levels and improving histopathological scores. In addition to lowering malondialdehyde levels, HES-SLN raised cardiac catalase and superoxide dismutase levels to levels that were similar to the control group. Additionally, caspase-3 levels were found to be much lower after HES-SLN therapy. Our findings suggest that HES-SLN may protect the heart from DOX-induced damage by reducing oxidative stress and cell death 66.

### Iron oxide-based nanomedicines in reducing cardiotoxicity

Iron oxide nanoparticles outperform other metal nanoparticles in terms of toxicity, surface reactivity, adsorption capacity, magnetic characteristics, and catalytic effectiveness 77. The reason behind this is their small size, superparamagnetic, and biocompatibility. Biological methods allow for the synthesis of three distinct iron oxide nanoparticles with intriguing characteristics by varying the reaction conditions 78. Due to the unique features of iron and nanosize, iron nanoparticles have been produced as a novel technology and display remarkable efficiency in applications across various fields. The synthesis of zero-valent nanoparticles often involves physical and chemical techniques. Oxidation occurs easily when exposed to air and humidity. Surface modification materials, loaded composites, and bimetallic techniques are utilised to prepare functionalized iron nanoparticles. Biologically functionalized iron nanoparticles are synthesised using a variety of microbes, including plants, bacteria, algae, and fungi 79. Stabilisers are optionally added during the production of bimetallic iron nanoparticles. A variety of stabilisers, both organic and inorganic, can be used to alter iron nanoparticles. Due to their potential applications in fields such as nanotechnology, medicine, and electronics, magnetic nanoparticles have garnered a lot of interest. Their many applications include medication administration, imaging contrast, and colloidal mediation in magnetic hyperthermia cancer treatments 80. Nanomedicine based on iron oxide show potential in reducing the cardiotoxicity caused by pharmacological formulations. With their controlled drug release capabilities, this nanomedicine can ensure targeted distribution to cardiac regions while minimising systemic exposure 81. Through the use of guided delivery and imaging made possible by their magnetic characteristics, accurate localization, and monitoring are made possible. Additionally, iron oxide nanoparticles have antioxidant characteristics that mitigate the oxidative stress that is associated with cardiotoxicity 82. In addition, the fact that they break down naturally reduces worries about their toxicity and accumulating over time. Nanomedicine based on iron oxide offer a multi-pronged strategy to reduce cardiotoxicity, which could improve the efficacy and safety of cardiovascular treatments by integrating drug delivery, imaging, and antioxidative effects 83.

**Figure 3:**
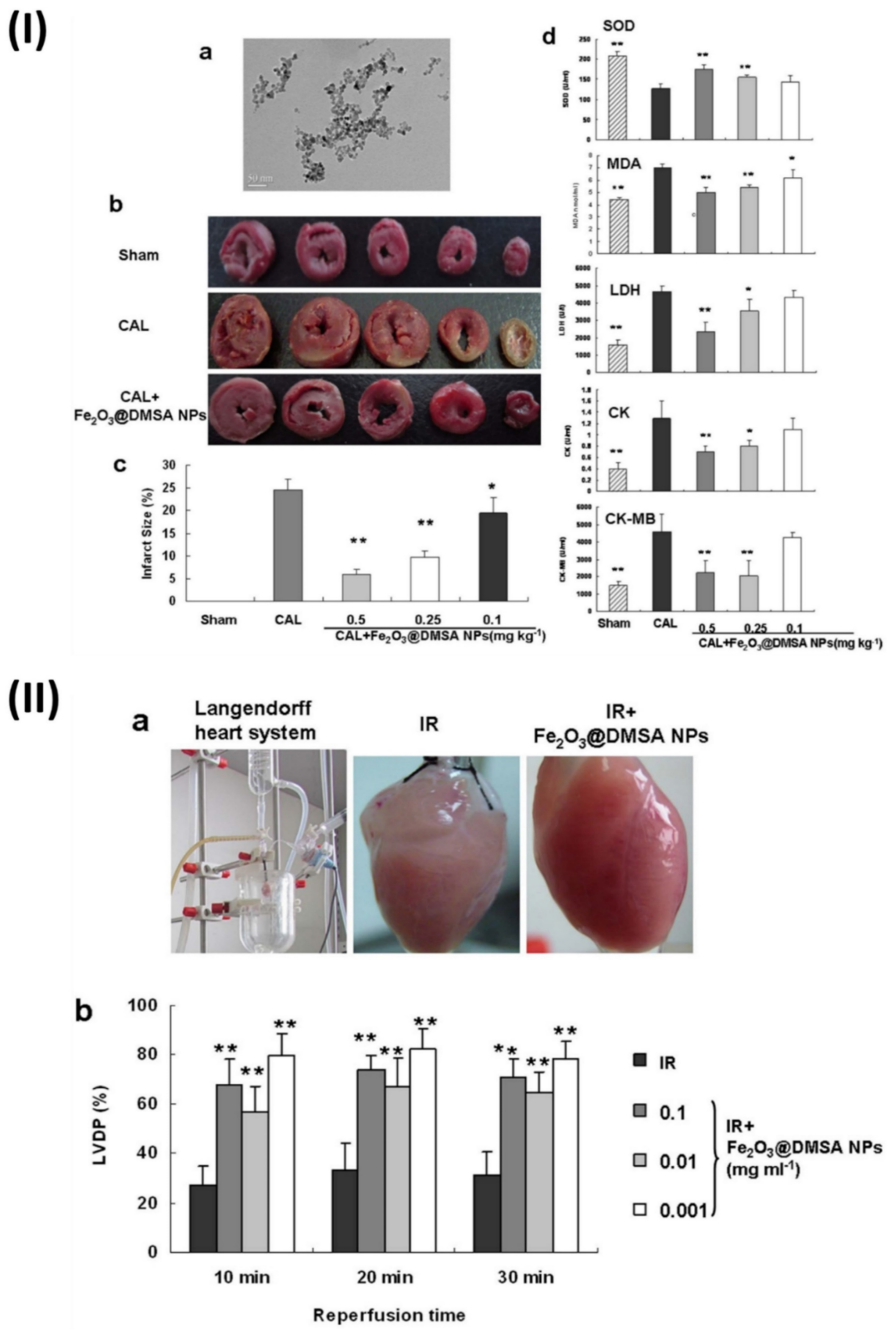
(**I**) Preventing coronary artery ligation (CAL)-induced damage in rats with Fe2O3@DMSA NPs; (**II**) The Langendorff heart of guinea pigs show cardioprotective effect after 30 minutes of reperfusion and 30 minutes of ischemia when treated with Fe2O3@DMSANPs. Reproduced with the permission from ref. 67. Fig. 1, and Fig. 2 (Springer©2015).

In a study, Xiong *et al.* found that iron oxide (Fe_2_O_3_) NPs can prevent ischemia injury to hearts on multiple levels. It is not the surface charges or molecules incorporated into nanoparticles that are responsible for their cardioprotective action, but rather the integrity of the nanoparticles themselves. In addition, normal cardiomyocytes were not significantly damaged by Fe_2_O_3_ NPs, suggesting that these nanoparticles may have therapeutic use in the treatment of cardiovascular disorders. A spherical core with an average diameter of 9.8 nm was observed in the produced NPs by transmission electron microscopy. To examine the possible protective effects of NPs on the heart, researchers used a rat coronary artery ligature (CAL) model to study NPs effects on myocardial infarct size and biochemical indices. Each Sprague-Dawley rat was given an injection of either a normal saline solution (CAL group) or (Fe_2_O_3_@2, 3-dimercaptosuccinic acid NPs) (Fe_2_O_3_@DMSA NPs) (CAL1Fe2O3@DMSA NPs group, 0.1, 0.25, 0.5 mg Fe kg) once a day via the tail veins to CAL surgery injury induction. After 30 min after injury, the infarct regions of the rats treated with Fe2O3@DMSA NPs were much less compared to the rats treated with normal saline. At 0.5 mg/kg, the Fe_2_O_3_@DMSA NPs-mediated enhancement peaked, but it was also dose-dependent. Superoxide dismutase (SOD), malondialdehyde (MDA), lactate dehydrogenase (LDH), creatine kinase (CK), and creatine kinase isoenzyme-MB (CK-MB) levels were among the biochemical indices determined in serum to confirm the protection. Compared to rats given normal saline, animals treated with Fe_2_O_3_@DMSA NPs at doses of 0.5 and 0.25 mg kg exhibited much greater SOD activity. Additionally, when compared to rats given with normal saline, rats treated with Fe_2_O_3_@ DMSA NPs had much reduced levels of MDA, LDH, CK, and CK-MB, as well as lower MDA activities and content. These findings proved that, in animal models, Fe_2_O_3_@DMSA NPs prevented myocardial damage due to ischemia. A commonly utilised tissue-level model for evaluating prospective cardiovascular medicines, the Guinea pig Langendorff heart, was produced to confirm the cardioprotective efficacy of Fe_2_O_3_@DMSANPs. The hearts that had been perfused were put through ischemia and reperfusion either with or without Fe_2_O_3_@DMSANPs. During reperfusion, the heart rate was unaffected by the therapy with Fe_2_O_3_@DMSANPs. A notable improvement in the left ventricular developed pressure was observed in the hearts treated with Fe_2_O_3_@DMSA NPs at concentrations ranging from 0.001-0.1 mg/ml within 30 minutes of reperfusion, in contrast to the hearts that were not treated. Additionally, the hearts that were treated with Fe_2_O_3_@DMSA NPs appeared redder and more normal in colour compared to the untreated hearts (Fig. 3) 67.

To image and treat C26 cells, which are target cells of murine colon cancer, Jalalian *et al.* examined the Epirubicin-5TR1 aptamer-SPION tertiary complex. Epi or Epi-Apt-SPION tertiary complex were administered to C26 and CHO-K1 (Chinese hamster ovary cells, nontarget) cells for cytotoxic investigations (MTT assay). Flow cytometry was used to assess internalisation. Lastly, *in vivo* imaging of cancer was performed using the Apt-SPION bioconjugate. The tertiary complex was efficiently internalised by C26 cells, according to flow cytometric measurements, but not by CHO-K1 cells. Results showing cytotoxicity of the Epi-Apt-SPION tertiary complex corroborated the data on internalisation as well. As compared to Epi alone, the combination exhibited reduced cytotoxicity in CHO-K1 cells. The viability of C26 cells treated with either Epi or complex did not differ significantly. Results from the magnetic resonance imaging (MRI) showed that the nanomagnets had accumulated heavily at the tumour site. In addition, this compound has the potential to limit tumour growth *in vivo* and effectively detect tumours when analysed by MRI 68.

### Micelle nanomedicines in reducing cardiotoxicity

Amphiphilic colloidal formations with a particle width ranging from 5 to 100 nm are known as micelles. Micelles are made up of molecules that have two halves that have distinct water affinities. Micelles are formed when amphiphilic molecules bind together at specific concentrations and temperatures. When micelles start to aggregate and form, this concentration is called the crucial micelle concentration 84. Micellar molecules combine at the crucial micellar temperature, but do not form micelles below this point; at this temperature, they remain monomers. The aggregation number of a micelle is the measure of how many monomer molecules make up the micelle. The production of micelles, which are aggregates of amphiphilic molecules, happens when the hydrophobic micelle fragments are removed from the water and hydrogen bonds are formed in the water, resulting in a decrease in the system's free energy 85.

A potential approach to reducing cardiotoxicity in medication formulations is the use of micelles nanoparticles. Encapsulating cardiotoxic medicines within their hydrophobic cores, these self-assembled structures are made of amphiphilic molecules 86. By decreasing off-target effects on the heart and limiting systemic exposure, this encapsulation allows controlled drug release. Micelles can also have their surfaces modified in a way that allows them to be delivered specifically to cardiac tissues, which can increase the therapeutic efficacy and decrease the side effects. Their diminutive stature allows for more effective cellular absorption, which in turn aids in the delivery of drugs to the heart. When it comes to increasing the safety and efficacy of cardiovascular medicines, micelles nanoparticles offer a diverse and effective technique for addressing cardiotoxicity 87.

**Figure 4:**
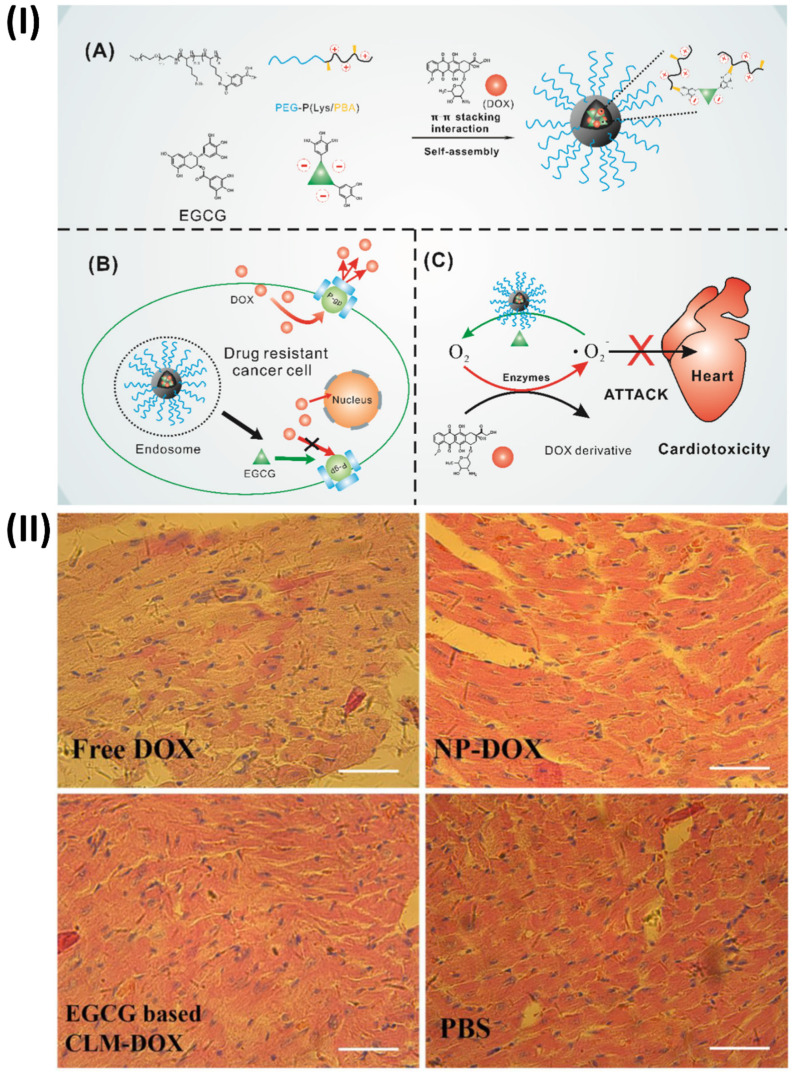
(**I**) (**A**) Diagram showing the process of creating a core cross-linked micelle that is co-loaded with DOX. (**B**) Developing a strategy to combat resistance to multiple drugs. (**C**) Approach to mitigate cardiac damage, (**II**) Examination of the histopathology following H&E staining of hearts obtained from mice administered free DOX, NP-DOX, CLM-DOX, or saline. Reproduced with the permission from ref. 69. Fig. 1, and Fig. 8 (Theranostic©2016).

An innovative micelle containing doxorubicin (DOX) and (-)-Epigallocatechin-3-O-gallate (EGCG) was prepared by Cheng *et al.* combined EGCG with poly (ethylene glycol)-block-poly (lysine-co-lysine-phenylboronic acid) (PEG-PLys/PBA) through electrostatic interaction and phenylboronic acid-catechol interaction. Via π-π stacking interaction with EGCG, DOX was co-loaded into the PIC micelles. Because of the interaction between phenylboronic acid and catechol, the PIC micelles are very stable in physiological conditions. When EGCG and DOX are delivered to the same site, they have synergistic effects due to the acid cleavability of the phenylboronic acid-catechol interaction in the micelle core. Further, histopathologic evaluation of hearts suggests that cardiomyocytes could be protected from DOX-mediated cardiotoxicity by combining EGCG with DOX in the micelle core, taking advantage of EGCG's oxygen free radical scavenging capacity. As a result of EGCG's regulation of P-glycoprotein (P-gp) activity, these PIC micelles have the potential to undo cancer cells' multidrug resistance. These findings provided strong evidence that PIC micelles derived from EGCG may successfully counteract DOX-induced cardiotoxicity and multidrug resistance (Fig. 4) 69.

## Nanotheranostics of anticancer drug induced cardiac toxicity

### Polymer-based nanotheranostics of anticancer drug-induced cardiac toxicity

A promising new field of nanomedicines based on polymers is blooming right now. One such field is polymer-drug conjugates (PDCs), which are nanoparticles that have garnered a lot of interest in the last decade 88. The chemistry of the pharmaceuticals and the polymers determines the specific chemical pathways that are used to manufacture PDCs, which are a unique type of macromolecules. Nanotheranostics made of polymer-drug conjugates are the subject of active research involving a wide range of polymers 89. Their potential for use in nanotheranostic applications is enhanced by the fact that the polymer chain contains free functional groups that can be linked with various agents, including imaging, targeting, and therapeutic moieties 90. When making PDCs, a combination of natural and synthetic polymers is utilised. Polymeric theranostic agents comprise four essential components: an imaging moiety, a medication, a coating to enhance biocompatibility and stability, and functional moieties to target cells 91. Multiple imaging, diagnostic, and targeting modes define polymer-based theranostic agents. The potential of polymeric-based nanotheranostics to integrate therapeutic and diagnostic functions is exciting since it could help alleviate cardiac toxicity caused by anticancer drugs. Biocompatible polymer nanoparticles encase anticancer medications, allowing for regulated release to reduce cardiotoxicity 92, 93. In addition, they have imaging agents that allow for the tracking of medication distribution and heart function in real-time. By modifying the surface, it is possible to minimise off-target effects and maximise targeted delivery to cardiac regions. Even more impressively, these nanotheranostics have antioxidative qualities that mitigate the oxidative stress linked to heart damage. With their versatile architecture, polymeric-based nanotheranostics provide a thorough solution for controlling cardiac toxicity caused by anticancer drugs. This improves the effectiveness of treatment while also ensuring the safety of patients 94.

In a study, Afrin *et al.* prepared a fluorescent nanoprobe to detect anticancer medication toxicity in heart tissue. Due to inflammation in the epithelial cells, they found that doxorubicin (DOX) treatment resulted in an overexpression of the vascular cell adhesion molecule 1 (VCAM1) protein and collagen in the heart of animals. They postulate that the afflicted heart can be detected and visualised by creating a fluorescent nanoprobe based on a VCAM1-targeted peptide (VHPKQHRGGSKGC). To address this, they synthesised a PLGA nanoparticle that was fused with the VCAM1 peptide and rhodamine B (RhB). They employ RhB fluorescence dye to see the particles and the tissue they're linked to. known as PLGA-VCAM1-RhB. In contrast to the untreated cells, DOX-treated human cardiomyocyte cells (HCMs) exhibited selective binding and a greater accumulation of the PLGA-VCAM1-RhB nanoprobes. A total of three injections of DOX (5 mg/kg) were administered via the tail vein every two weeks for six weeks in the *in vivo* trials. After one week of the last dose of DOX, PLGA-VCAM1-RhB and PLGA-RhB were injected into the tail vein, and pictures were taken four hours after the injection.

**Figure 5:**
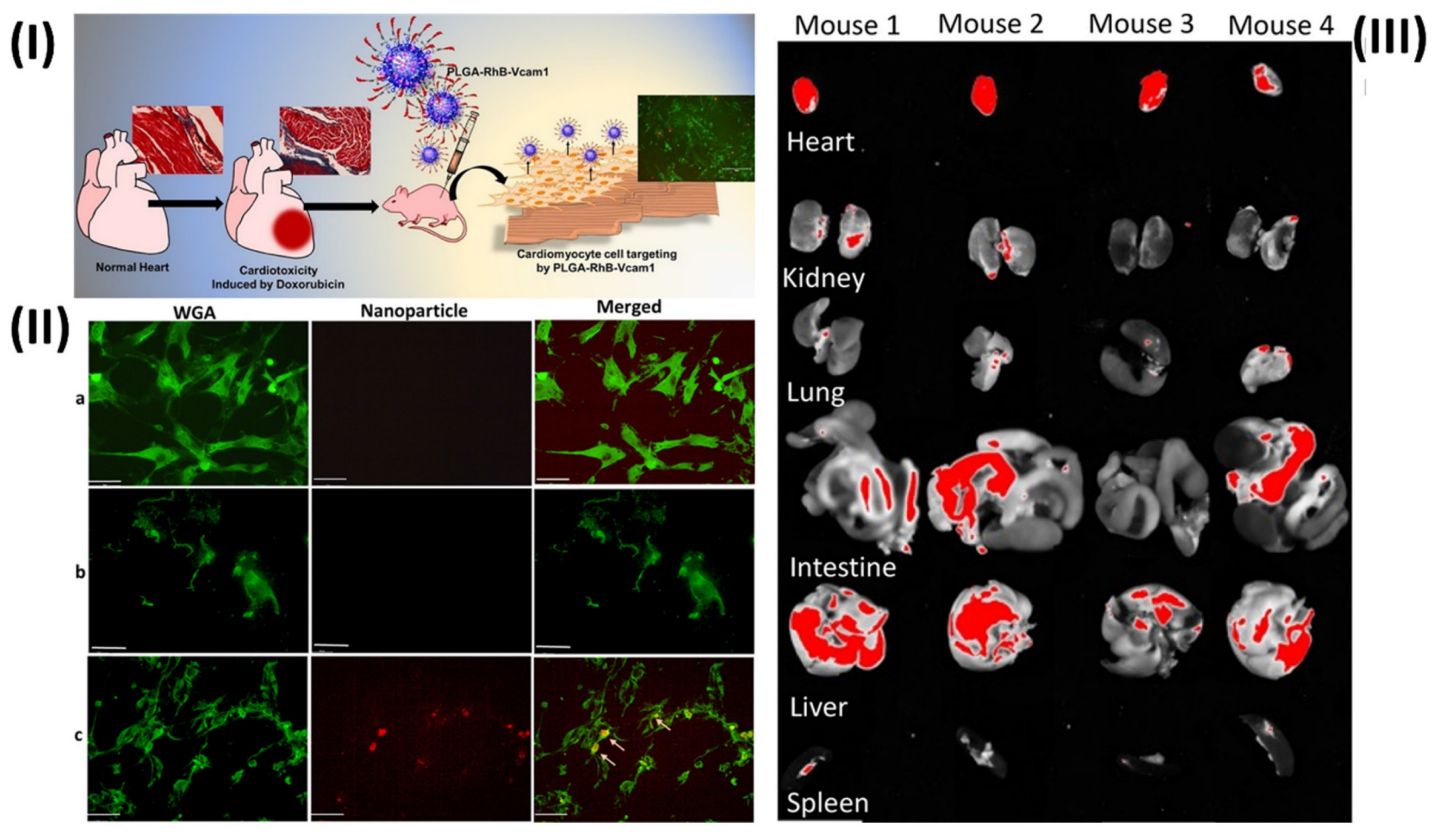
(**I**) Graphical abstract shows the cardiotoxicity induced by anticancer drugs and the effects of nanotheranostics; (**II**) Microscopic image showing the uptake of nanoparticles in human cardiomyocyte cells (HCMs); (**III**) Deposition of the particle (PLGA-RhB-VCAM1) in other major organs. Reproduced with the permission from ref. 95. Graphical Abstract, Fig. 6, and Fig. 7 (ACS Publication©2022).

The specificity and targeting capacity of PLGA-VCAM1-RhB were demonstrated by the increased fluorescent signal of PLGA-RhB-VCAM-1 (48.62% ± 12.79%) in the heart of DOX-treated mice compared to the untreated control group (10.61% ± 4.90) through the irritated tissues. In comparison to the healthy control group, the homogenised heart tissue of PLGA-RhB-VCAM1 exhibited a fluorescence intensity that was 156% greater when quantified. The potential for PLGA-VCAM1-RhB to bind inflammatory cardiac cells allows us to conclude that it can detect DOX-induced cardiotoxicity and damaged heart at an early stage. A study comparing the uptake of targeted nanoparticles VCAM1 (PLGA-RhB-VCAM1) with that of nontargeted particles (PLGA-RhB) was carried out in cells that were treated for 72 hours. Based on the results, PLGA-RhB tends to accumulate in the cells without any particular purpose, although it is most commonly seen outside of cells (Fig. 5II). Conversely, the cells were able to absorb and use the PLGA-RhB-VCAM1 nanoparticle, which implies that the inflamed cell can bond with the VCAM1 peptide-linked particle and may be able to identify the area of myocardial injury. Fig. 5IIIA shows that the buildup of PLGA-RhB-VCAM1 was also examined using pictures of other significant organs. They used ImageJ to calculate the percentage of fluorescent area and the mean intensity of RhB in the photos. In the heart, the mean intensity was 190.32, while in the liver it was 154.22, the lungs 92.2, the kidneys 88.79, the spleen 53.87, and the intestines 118.46. In the aforementioned organs, assessing the area covered with fluorescence RhB did not reveal any substantial accumulation of particles 95.

**Figure 6:**
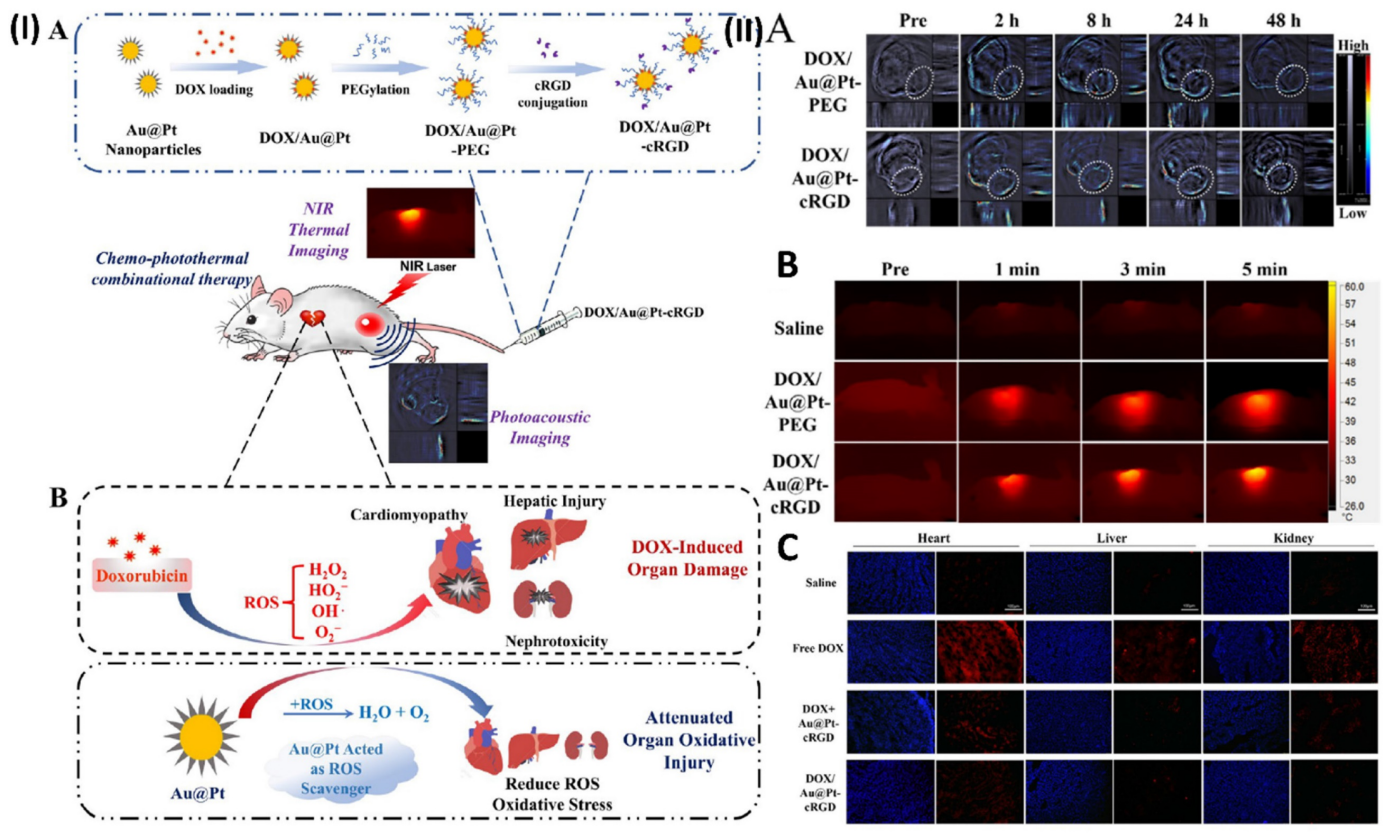
(**I**) (**A**) Development of DOX/Au@Pt-cRGD; (**B**) The ability of nanomedicine to reduce DOX-induced organ oxidative damage, particularly cardiomyopathy during chemotherapy, is a result of its ROS scavenging capabilities, which are achieved through the catalysis of platinum shells. (**II**) Using Au@Pt to direct imaging *in vivo*. (**A**) Pre- and post-intravenous injection of DOX/Au@Pt-cRGD and DOX/Au@Pt-PEG to tumor-bearing animals undergoing PA imaging *in vivo*. (**B**) *In vivo* photothermal processing of DOX/Au@Pt-cRGD (C) Image of heart, liver, and kidney tissue treated using various fluorescent microscopy techniques. Reproduced with the permission from ref. 96. Scheme 1, Fig. 5, and Fig. 8 (ACS Publication©2018).

In another study, Yang *et al.* construct porous Au@Pt nanoparticles and investigate their potential for chemo-photothermal co-therapy to limit tumour growth and alleviate oxidative stress damage. Doxorubicin (DOX) can be loaded into the Au@Pt nanoparticles through their adjustable surface pore structure. DOX-loaded Au@Pt nanoparticles with enhanced drug delivery capabilities are made possible by modifying the cRGD peptide. A regulated release of the drug is seen in the nanocarrier (DOX/Au@Pt-cRGD). Au@Pt nanoparticles are an ideal vehicle for photothermal therapy (PTT) due to their high photoconversion efficiency and the fact that their structure has an absorbance peak in the near-infrared (NIR) region, which allows for *in vivo* photoacoustic imaging. The nanosystem DOX/Au@Pt-cRGD demonstrates increased anticancer therapeutic benefits when combined with chemotherapy. Crucially, the ROS-scavenging activity of Au@Pt reduces the damage caused by oxidative stress in the presence of DOX, which is particularly helpful in preventing cardiomyopathy that might occur after chemotherapy. To reduce adverse effects associated with chemo-photothermal combination therapy, this DOX/Au@Pt-cRGD nanosystem could be investigated as a reactive oxygen scavenger and medication delivery system. One group of subcutaneous MDA-MB-231 tumor-bearing cells was given DOX/Au@Pt-cRGD (10 mg/kg) while another group received DOX/Au@Pt-PEG (10 mg/kg) intravenously. At various time intervals (pre-injection, 2, 8, 24, and 48 h after tail-vein injection, Fig. 6IIA), PA imaging and PA signal intensity of the tumour inside Balb/C nude mice were recorded, respectively. Furthermore, they conducted a more thorough evaluation of the DOX/Au@Pt-cRGD accumulation in the tumour xenograft using infrared thermal imaging. Different groups of mice were injected intravenously with DOX/Au@Pt-cRGD and DOX/Au@Pt-PEG, demonstrating that Au@Pt nanoparticles are a potential choice for photothermal therapy. The measurements of the tumour site's surface temperature were taken down (Fig. 6IIB). After 5 minutes of irradiation, the tumour surface temperatures of the DOX/Au@Pt-cRGD group reached 60 °C, a much higher rate than that of the DOX/Au@Pt-PEG groups (51 °C in 5 minutes). The data also showed that after modifying cRGD, the tumour location had an elevated enrichment of Au@Pt nanoparticles. The production of ROS generated by DOX was determined by DHE staining. Superoxide anions oxidise DHE, causing it to exhibit red fluorescence in the cytosol. The control group that did not receive any DOX showed a substantial increase in DHE fluorescence intensity, which may indicate an excess of ROS. Nevertheless, the DHE fluorescence intensity was considerably reduced following the injection of Au@Pt-cRGD with DOX, indicating an inhibitory effect on oxidative stress. Fig. 6IIC shows that the DOX/Au@Pt-cRGD treated group had the best antioxidative effect since they had the lowest level of DHE fluorescence 96.

### Carbon-based nanotheranostics of anticancer drug-induced cardiac toxicity

Carbon is a unique material with many diverse qualities; it is also the basis of one area of nanotechnology. As a nanomedicine with promising prospective uses in nanomedicine, bioelectric technology, and technology, carbon in its most common allotropes appears to be quite biocompatible with a wide range of agents and has very low cytotoxic effects 97. An understanding of the fundamental mechanical, electrical, optical, and magnetic characteristics of nanomedicine can lead to the development of novel functional materials with several potential uses in the expanding field of nanotechnology 98.

**Figure 7:**
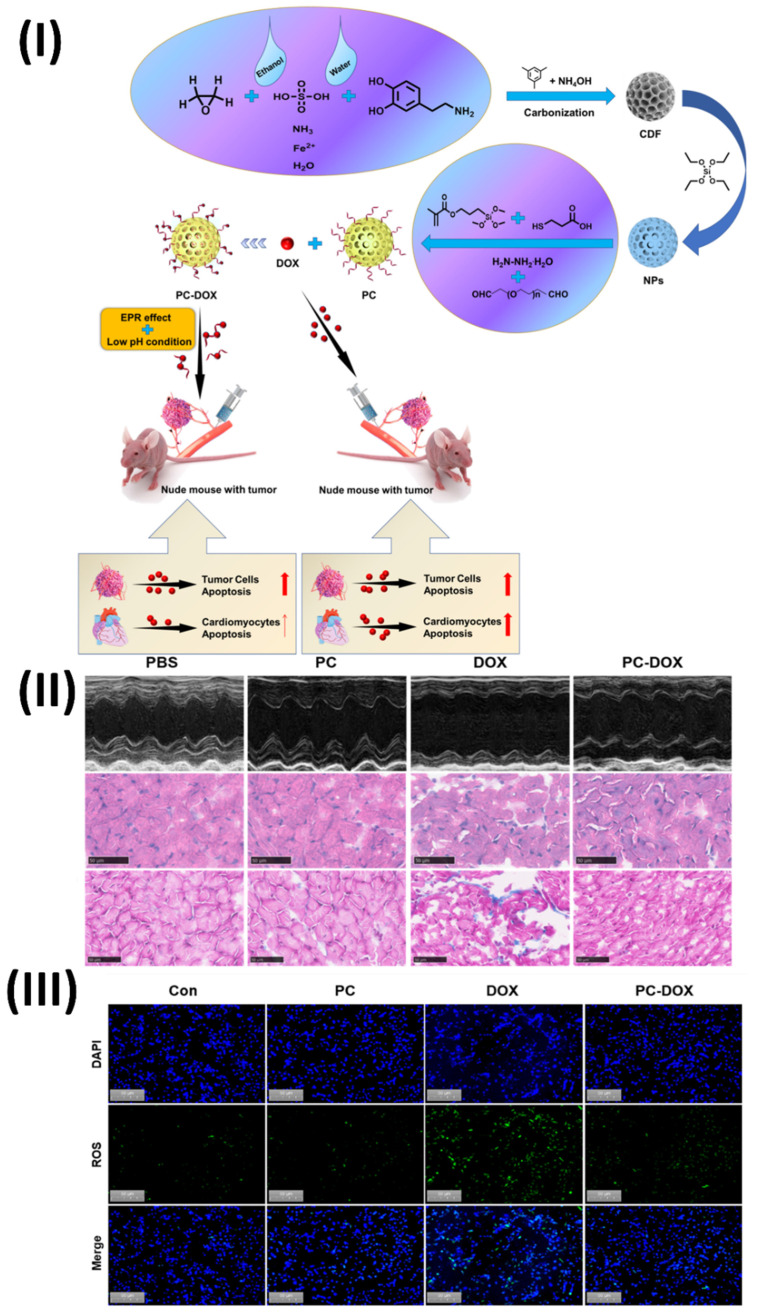
(**I**) Diagram depicting the process of PC-DOX production, treatment for tumors, and mitigation of cardiotoxicity; (**II**) DOX-induced cardiac damage is reduced by PC-DOX. (a) Left ventricular morphologies as seen in representative M-mode echocardiograms from each category; (**III**) Illustrations of representative DCFH-DA *in vitro* cell populations. Reproduced with the permission from ref. 99. Fig. 1, Fig. 7, and Fig. 8 (Elsevier©2023).

In a study, Wang *et al.* constructed a pH-responsive imine bond-containing carbon-based drug delivery system (PC-DOX) that loaded doxorubicin. For the synthesis of PC-DOX, which has a consistent particle size of about 180 nm, dialdehyde PEG was applied to as-synthesized hollow carbon-based NPs. The NPs served as a carrier for the covalent attachment of DOX through a dynamic covalent connection. Because of its one-of-a-kind structure, PC-DOX can deliver drugs specifically to tumour microenvironment (TME) and targets tumours. One positive aspect of the EPR effect is that it has the potential to boost tumour cellular uptake through tumour targeting. On the flip side, pH-responsiveness may allow for efficient DOX accumulation in tumour tissues while normal tissues are spared undesirable side effects. Consequently, PC-DOX had a notable anti-tumor impact at a low mass concentration (DOX equivalent dose: 20 μg/mL) in addition to a high DOX loading capacity (70.12%) and outstanding biocompatibility. *In vitro* cellular and animal tests demonstrated PC-DOX's outstanding protective activity against DOX-induced cardiotoxicity, which was another appealing feature. On average, cardiomyocyte viability increased by 30.58% when compared to free DOX, and cardiac function was also noticeably enhanced. This innovative drug delivery nanoplatform opens up new possibilities for the therapeutic use of DOX in cancer treatment in the future. The efficacy of PC-DOX in ameliorating DOX-induced cardiac dysfunction was assessed using echocardiography. The results show that the mice in the PC-DOX group had significantly higher left ventricular ejection fraction (EF) and fractional shortening (FS) than the free DOX group (Fig. 7II). The left ventricular end-diastolic dimension (LVEDD) and left ventricular end-systolic dimension (LVESD) were both considerably reduced at the same time. The lack of fluorescence in the control and PC groups was seen in Fig. 7III. In contrast, free DOX caused substantial oxidative stress in the cells, as shown by the bright green fluorescence seen in the DOX-treated group. The PC-DOX-treated group did show some green fluorescence, but it was much weaker than in the free DOX group. Based on these results, PC-DOX may be able to reduce DOX-induced oxidative stress in the hearts by inhibiting DOX-induced oxidative stress in H9C2 cells. Combining therapeutic and diagnostic qualities, carbon-based nanotheranostics offer a potential technique for treating cardiac toxicity caused by anticancer drugs. To reduce the risk of cardiotoxicity, anticancer medications are encapsulated in this nanomedicine, which are often carbon nanotubes or graphene. Their large surface area makes them ideal for loading and delivering drugs to heart tissues.

Additionally, imaging agents are included with carbon-based nanotheranostics to provide early toxicity identification by real-time monitoring of heart function and drug distribution. By making it easier to target the heart, functionalization helps to decrease off-target effects during delivery. Nanotheranostics derived from carbon provide a holistic strategy for reducing cardiac toxicity caused by anticancer medications, which in turn improves treatment results, owing to their multifunctional properties 99.

### Micellar system-based nanotheranostics of anticancer drug-induced cardiac toxicity

One popular and effective way to overcome the problems of drug delivery is the nanomedicine technique, which is being used more and more for hydrophobic medication delivery. To accomplish the intended therapeutic effect, it is necessary for a sufficient amount of the active medication to reach the site of action and maintain an effective concentration there for a specific duration 100. The great majority of medications, however, have this process impeded for various reasons. Some of these difficulties include systemic toxicity, insufficient targeting of specific tissues, fast drug breakdown *in vivo*, and poor pharmacokinetics (PK). Nanoparticulate medications, such as micelles based on polymers, have been used to encapsulate pharmaceuticals 101. The use of nanomedicines holds great promise for achieving both localised accumulation and sustained circulation. It is possible to create polymeric micelles using either natural macromolecules or biocompatible synthetic polymers 102. The shape, stability, drug-loading capacity, and drug-release profile of the polymeric micelle are mostly dictated by the core-forming segments 103. Due to their hydrophilic polymeric coating, they will be able to avoid detection by the RES while in circulation. The hydrophilic micelle shell improves the nanoparticles' steric stability and decreases their non-specific absorption by the RES 104. This causes the blood to remain in circulation for a longer duration. Micelles must be able to circulate steadily in the blood compartment and avoid unwanted interactions with blood components and the RES for their delivery to be successful. In addition, micelles should extravasate selectively at the site of illness (tumour), where they can be taken up by the target cells and released intracellularly 105. The quick and even distribution of micelles throughout the body is made possible by injecting them directly into the bloodstream. Micellar formulations are delivered for a variety of localised non-cancer disorders using alternative modes of administration, including oral, transmucosal, or topical administration. Nanotheranostics based on micellar systems offer a potential solution to the problem of anticancer drugs' effects on the heart through their combined diagnostic and therapeutic capabilities 106. Encapsulating anticancer medications for controlled release, these nanomedicines which consist of self-assembled micelles of amphiphilic molecules minimize cardiotoxicity. By integrating imaging agents, heart function and medication distribution can be monitored in real-time, allowing for the early diagnosis of toxicity. Targeted distribution to cardiac tissues is made possible through surface changes, minimising off-target effects. Additionally, antioxidative characteristics of micellar nanotheranostics reduce oxidative stress linked to heart damage 107.

**Figure 8:**
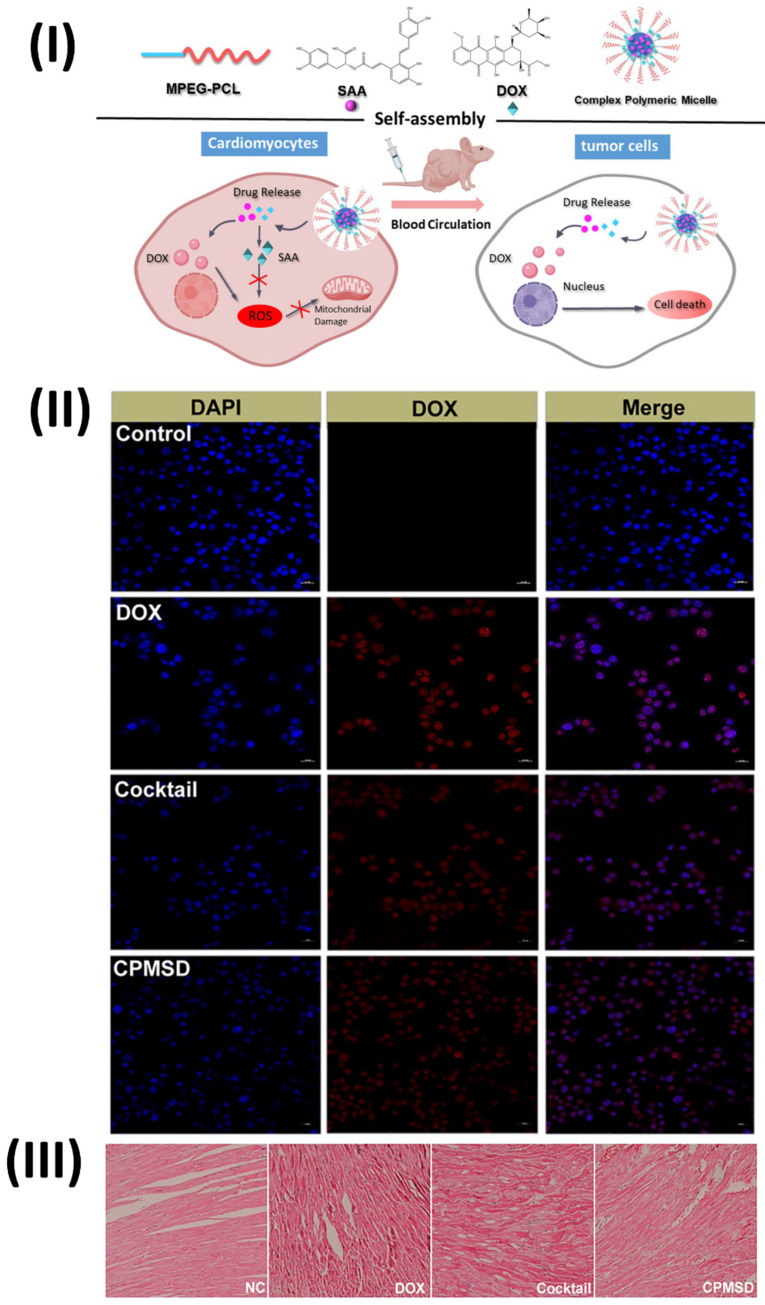
(**I**) Diagram depicting the self-assembly and multi-function of CPMSD in cancer treatment; (**II**) Cellular uptake as observed by CLSM (III) Investigation using a light microscope. Reproduced with the permission from ref. 108. Fig. 1, Fig. 6, and Fig. 9 (Springer©2022).

In a study, Li *et al.* developed a complex polymeric micellar system called Compound Micelles of DOX and SAA (CPMSD). The DOE investigation yielded the optimal formulation, and the CPMSD micelles were successfully generated by entrapping the drug-carrier mass ratio at 1:5 and the DOX-SAA mass ratio at 1:4. The results of the molecular dynamics simulation showed that SAA was co-encapsulated in the hydrophobic cavity with DOX, while the latter was bound to the micelle's ball-shaped surface because of its hydrophilicity. The characterization investigation revealed some promising biopharmaceutical qualities, including small and uniform particle size, decent colloidal stability, regulated drug release, and a pretty high drug loading capacity. No change was observed in the anticancer efficacy or action mechanism of DOX when SAA was co-administered with CPMSD. Importantly, it helped protect tumor-bearing mice's hearts from the oxidative stress damage caused by DOX and had a positive impact on systemic safety. Based on all of the results, CPMSD seems like a DOX nanosystem with several potential uses in the treatment of breast cancer. The *in vitro* growth of MCF-7 or MCF-10A cells was barely affected by SAA alone at a final concentration of 10 μM, and the relative inhibition rate was less than 20% (Fig. 8II), suggesting that SAA is not a cytotoxic agent like DOX. Consistent with earlier reports on the biological and pharmacological activities of this natural product, the results indicate that combining SAA with DOX, which has different action mechanisms, could be an effective comprehensive treatment strategy for breast cancer. Cardiomyocyte morphology in the left ventricle of tumor-bearing nude mice in the NC group was essentially normal, as shown in Fig. 8III. However, cardiomyocyte morphology could be affected in varying ways and to varying degrees by DOX-containing treatments, with the most severe myocardial damage seen in mice treated with free DOX. Along with the presence of inflammatory cells, the group that received only DOX showed clear signs of myocardial injuries, including cross-striations, puffing of the myocardial endochylema, and partial resorption of the sarcoplasmic matrix. Additionally, there was a disarray of the myocardial fibres, swelling of the cells, and degeneration, all of which could be explained as toxin-mediated necrosis of the cardiomyocytes. Specifically, the CPMSD micelles showed fewer histopathological alterations than the cocktail formulation at the same dosage, suggesting that co-administration of SAA with DOX may mitigate this cardiomyocyte damage. Collaborative administration of SAA and DOX via the CPMSD formulation significantly reduced the cardiotoxic effects of DOX, as shown by all of these results 108.

### Exosome-based nanotheranostics of anticancer drug induced cardiac toxicity

Exosomes are released into the bloodstream and other bodily fluids by all cells, carrying cargo from their host cells 109. This makes them potential biomarkers for certain disorders. Paracrine, autocrine, and even endocrine signalling pathways are additional ways by which exosomes might influence host or recipient cells 110. A major limitation of EVs research, despite its abundance of literature, is the absence of standardised techniques for isolation, especially when it comes to human bodily fluid exosomes. Additionally, the produced EVs still contain impurities such as big vesicles and proteins 109. Hence, the effectiveness of separating the pure form of EVs is a crucial component in establishing their functional and molecular capacities, which are relevant when thinking about therapeutic experimental studies and downstream biomarkers. The novel characteristics of exosome-based nanotheranostics make them an attractive candidate for the management of cardiac toxicity caused by anticancer drugs 111. Therapeutic and diagnostic substances can be carried by these nanoparticles in exosomes, which are natural vesicles released by cells. With their controlled medication release, exosomes reduce cardiotoxic effects and maximise the efficacy of anticancer treatments. In addition, ligands that target the heart can be engineered into exosomes, allowing for targeted delivery to the heart while minimising side effects. Furthermore, exosomes can be used to evaluate cardiotoxicity in real-time since they include endogenous proteins that allow imaging of heart function and drug distribution 112. One potential approach to reducing anticancer drug-induced heart toxicity and enhancing treatment results is the use of exosome-based nanotheranostics, which possess both natural biocompatibility and multifunctionality 113.

**Figure 9:**
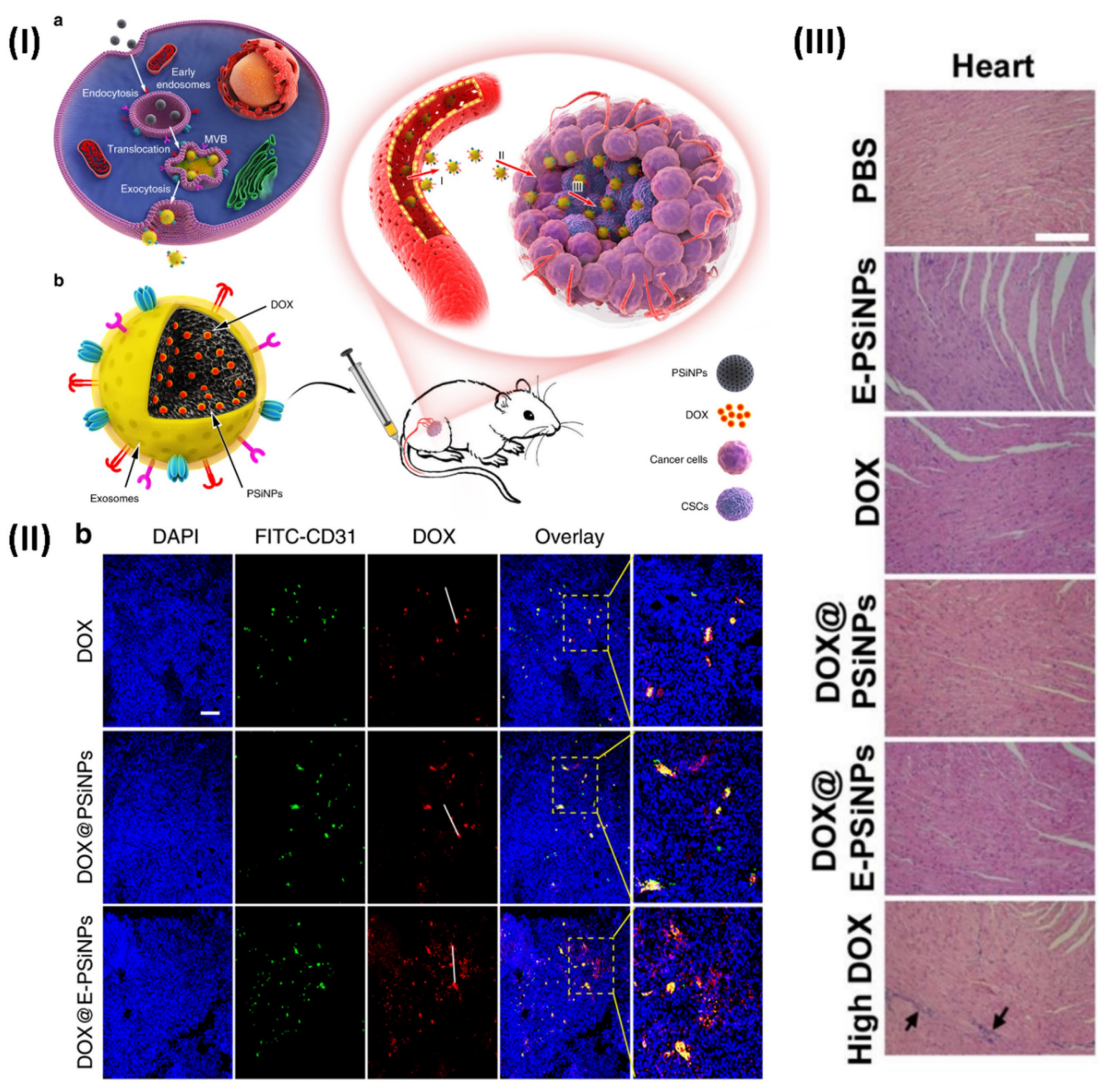
(**I**) An illustration depicting the use of E-PSiNPs as a vehicle for targeted cancer chemotherapy; (**II**) At 24 hours after receiving an intravenous injection of DOX, DOX@PSiNPs, or DOX@E-PSiNPs at a dosage of 0.5 mg kg-1, DOX and CD31-labeled tumor vasculature were observed colocalizing in tumor sections of mice containing the H22 tumor; (**III**) After being intravenously injected with PBS, E PSiNPs, free DOX, DOX@PSiNPs, or DOX@E PSiNPs at a dosage of 0.5 mg/kg, or free DOX at a dosage of 4 mg/kg once every three days for five times, the primary organs of mice having H22 tumors were stained with H&E. Reproduced with the permission from ref. 114. Fig. 1, Fig. 6, and Fig. S.30 (Springer Nature©2019).

In a study, Young *et al.* formulated biocompatible tumor-cell-exocytosed exosome-biomimetic porous silicon nanoparticles (PSiNPs). Intravenous delivery of exosome-sheathed doxorubicin-loaded PSiNPs (DOX@E-PSiNPs) by tumour cells increases tumour formation, extravasation from blood arteries, and penetration into deep tumour parenchyma. The DOX@E-PSiNPs have a strong affinity for cancer cells and cancer stem cells (CSCs) and can cause cytotoxicity in both types of cancer cells. The anticancer efficacy and reduction of CSCs in subcutaneous, orthotopic, and metastatic tumour models are brought about by these properties of DOX@E-PSiNPs, which allow them to greatly enrich total tumour cells and side population cells with CSC characteristics *in vivo*. As a prospective drug carrier for efficient cancer chemotherapy, the results demonstrate the feasibility of using exosome biomimetic nanoparticles exocytosed from tumour cells. At 24 hours post-injection, confocal fluorescence microscopy pictures made it very evident that DOX@EPSiNPs were dispersed throughout the entire tumour section. The DOX@PSiNPs and free DOX co-localized more strongly with FITC-CD31-labeled endothelial cells, suggesting that they aggregated more near the blood vessels (Fig. 9) 114.

## Conclusion and Future Directions

In light of the current state of knowledge regarding the frequency of cardiotoxicity as a side effect of anticancer chemotherapy medicines, this article offers a thorough review of the topic. Among the many side effects of available anticancer drugs, cardiac toxicity is the most prevalent. Several processes contribute to the detrimental impacts on the cardiovascular system, including autophagy, apoptosis, elevated oxidative stress, and the suppression of cardiac contractile performance. Cardiomyocyte death has evolved as the leading cause of long-term irreversible cardiac dysfunction, despite numerous proposed molecular and cellular pathways that mediate the cardiotoxic action of anti-cancer medications. Anthracyclines, fluoropyrimidines, and alkylating medications all have these significant adverse effects. Because adult human hearts have a severely limited capacity to create new cardiomyocytes, damaged cardiomyocytes can't be effectively repaired. Anticancer drugs can cause cardiomyocyte mortality and lasting damage through a variety of biological pathways; consequently, it is urged that measures be developed to increase cardiomyocyte survival to decrease this cell death and its consequences. Additional research into tailored treatments and chemotherapy that target cardiomyocyte survival determinants is necessary. Cancer patients who have sustained irreversible damage from anticancer medications may potentially benefit from investigating how to modulate the myriad of variables and signalling pathways that have been demonstrated to stimulate endogenous cardiomyocyte proliferation in the context of cardiac regeneration techniques. It is possible to test the hypothesis that modulating some of these parameters can mitigate the long-term cardiotoxic effects of anticancer medications by increasing cardiomyocyte lifespan. The anticancer drugs' ability to work should be thoroughly assessed. There is considerable hope that nanomedicine and nanotheranostics may one day help in the battle against cancer while mitigating the side effects of anticancer drugs on the heart.

Utilising state-of-the-art imaging techniques including multimodal imaging such as MRI/CT, ultrasound/photoacoustic and nanoparticle-based contrast agents, nanotheranostics provide new avenues for the early diagnosis and monitoring of cardiac toxicity. This paves the way for prompt action and the creation of tailored treatment programmes. Nanotechnology and theranostic capabilities allow for the simultaneous delivery of anticancer drugs to particular parts of the body with minimal risk of cardiac side effects. By regularly monitoring drug distribution and heart function, clinicians can fine-tune dosing regimens and treatment strategies to limit the risk of cardiotoxicity and enhance anticancer efficacy. Designing nanomedicine to accumulate exclusively in tumour tissues allows for the improvement of anticancer medicine therapeutic indices without causing harm to healthy cardiac cells. When novel anticancer drugs with substantial anticancer power do not have a strong safety index, this presents a problem for the healthcare providers interacting with patients, notably cardiologists and oncologists. To reduce the development of later stage issues, it is vital to examine patients regularly for probable cardiac toxicity. To create effective cardioprotective methods, it is necessary to thoroughly investigate the mechanisms of cardiotoxicity in each medicine. Nanomedicine and nanotheranostics have also been combined to create a new way to image the heart damage caused by anticancer drugs. This novel method has a lot of potential for enhancing cancer therapy safety and patient outcomes by allowing for the early identification and accurate monitoring of adverse cardiac effects.

## Figures and Tables

**Table 1:** Illustration of cardio toxicity based clinical investigation of commonly prescribed anticancer drugs

Chemical moieties	Diseases	Recommended Dose	Cardiac complication	Clinical outcomes	Ref.
Doxorubicin	Breast cancer, small cell lung cancer	50-1000 mg/m^2^	Congestive heart failure	A clinical trial study found that at a cumulative dose of 550 mg/m2, 26% of participants developed CHF.	19
Daunorubicin	Acute lymphocytic leukaemia	360-1260 mg/ m^2^	Congestive heart failure	Daunorubicin demonstrated cardiac toxicity (10%) in child patients	50
Imatinib	Gastrointestinal stromal tumors	400-800 mg	Cardiomyopathy, cardiac toxicity	In the Imatinib treatment of 55 patients, only 1 patient showed heart failure and demonstrated limited cardiac toxicity of this drug	51
Sunitinib	Gastrointestinal stromal tumors and Renal cell carcinoma	50-75 mg	Myocardial infarction, Congestive heart failure	This study demonstrated 8% CHF events and 28% LVEF declines with sunitinib treatment	52
Sunitinib and sorafenib	Renal cell carcinoma	50 mg/d, and 800 mg/d	Cardiac event and ECG change	33.8% of patients followed the cardiac event and 40.5% experienced ECG changes.	53
Paclitaxel	Ovarian cancer, breast cancer, non-small cell cancer	175-200mg/ m^2^	Congestive heart failure	18% of the women experienced reversible CHF condition	54
Cyclophosphamide	Hematologic malignant neoplasm	180 mg/kg	Peri cardiomyopathy	28% of the patients showed CHF symptoms after 3 weeks of cyclophosphamide administration	55
5-fluorouracil	Colorectal cancer, breast cancer, head and neck cancer	1000 mg/ m^2^	Myocardium damage, ECG abnormalities and cardiotoxicity	Approx. 4 % of the patient followed the ECG abnormalities with the incidence of cardiotoxicity	56
Transtuzumab	Metastatic breast cancer	250 mg	Cardiac Dysfunction	3-7% of the patient demonstrated cardiac dysfunction with the alone treatment of trastuzumab	57
Mitoxantrone	Aggressive multiple sclerosis	120 mg/ m^2^	Denovo cardiotoxicity, decreased LVEF	Mitoxantrone showed well tolerated in recommended dose, but cardiotoxicity (14%) was evident	58

**Table 2:** Several nanomedicines show promise as possible therapeutic agents for reducing cardiotoxicity

Nanomedicine	Therapeutic agent	Size (nm)	Concluding remarks	Ref.
Liposomes	Doxorubicin	98 nm	Results were encouraging when liposomal doxorubicin was used alone or when free doxorubicin and free quercetin were administered together.	62
Liposomes	Doxorubicin	-	As a first-line treatment for MBC, Myocet enhances doxorubicin's therapeutic index by lowering grade 4 neutropenia and cardiotoxicity while maintaining antitumor activity that is equivalent to that of cyclophosphamide alone.	63
Solid lipid nanoparticles	Doxorubicin	96 nm	Research suggests that RGD-DOX-SLNs might be an effective new lipid carrier for breast cancer treatment, based on anticancer findings in both vitro and vivo.	64
Solid lipid nanoparticles	Resveratrol	271 nm	Res-SLN protects the myocardium and lessens DOX-induced cardiotoxicity in mice, indicating a therapeutic benefit.	65
Solid lipid nanoparticles	Hesperidin	175 nm	The findings suggest that HES-SLN may protect the heart from DOX-induced damage by reducing oxidative stress and cell death.	66
Iron oxide nanoparticles	-	9.8 nm	Fe2O3 NPs can cure cardiovascular problems since they do not cause any major harm to normal cardiomyocytes.	67
Iron oxide nanoparticles	Epirubicin	51 nm	By analyzing tumors using MRI, this compound may effectively detect malignancies and, *in vivo*, limit tumor development.	68
Micelles	Green Tea Catechin, Doxorubicin	130-140 nm	The results indicated that PIC micelles derived from EGCG may successfully counteract cardiotoxicity caused by DOX and multidrug resistance.	69
